# Formation and Microstructural Evolution of Ferritic ODS Steel Powders during Mechanical Alloying

**DOI:** 10.3390/ma16020765

**Published:** 2023-01-12

**Authors:** Krzysztof Nowik, Rafał Zybała, Zbigniew Oksiuta

**Affiliations:** 1 Institute of Mechanical Engineering, Faculty of Mechanical Engineering, Białystok University of Technology, Wiejska 45C, 15-351 Białystok, Poland; 2 Łukasiewicz Research Network—Institute of Microelectronics and Photonics, Al. Lotników 32/46, 02-668 Warsaw, Poland; 3 Institute of Biomedical Engineering, Faculty of Mechanical Engineering, Białystok University of Technology, Wiejska 45C, 15-351 Białystok, Poland

**Keywords:** mechanical alloying, ball milling, line profile analysis, whole powder pattern modelling, ODS steel, microhardness

## Abstract

Ferritic ODS steel elemental powder compositions with various Zr content (0.3–1.0 wt.%), ground in a Pulverisette 6 planetary ball mill, were extensively studied by X-ray diffraction line profile analysis, microscopic observations, microhardness testing and particle size measurements. A characteristic three-stage process of flattening the soft powders, formation of convoluted lamellae and, finally, formation of nanocrystalline grains was observed. In order to quantify the microstructural properties, expressed mainly in terms of crystallite size and dislocation density, a methodology for detailed and accurate microstructure analysis of nanosized and severely deformed materials was proposed by the Whole Powder Pattern Modelling (WPPM) approach. In the case of the proposed ODS alloy composition, the overlapping of Fe and Cr Bragg reflections makes the microstructure analysis certainly more complicated. The results showed that the microstructure of powders evolved towards the nanocrystalline state consisting of fine (diameter of ~15 nm) and narrowly dispersed domains, with extensive dislocation density exceeding $10^{16}$ m^−2^.

## 1. Introduction

ODS steels belong to the group of structural nuclear materials, with great potential for use in future fusion reactors, so their industrial importance is indisputable. The development of ODS alloys has historically been driven by several technologically important applications that required improved mechanical properties at elevated temperatures. This reinforcement can be achieved by the incorporation of oxide particles into the microstructure of alloys. A milestone in the field of ODS materials occurred when Benjamin (1970) developed a new process called mechanical alloying (MA), initially to combine Y_2_O_3_ with complex Ni-based superalloy matrices [1]. During MA, elemental or pre-alloyed powders are intensely blended with an oxide of interest, usually Y_2_O_3_ (alternatively Y-containing intermetallic compound can be used, e.g., Fe_2_Y [2]). This pioneering development was adopted for producing ODS alloys that were used for high temperature structural applications, mainly in aviation and energetics, including the structural parts of the tokamak. It was quickly realized that MA is also amenable to making a variety of other alloys, even unusual combinations of metals and ceramics. Oxide dispersions are exceptionally attractive due to the thermodynamic stability of most oxide phases, and because they are potent barriers to migrating grain boundaries and dislocations, causing strengthening of the metallic matrix. In addition, immense quantities of nano-scale oxides trap He in fine-scale bubbles, suppressing void swelling and protecting grain boundaries from He accumulation, providing excellent irradiation resistance [3].

The MA process is usually controlled in terms of particles’ size and microstructure evolution by such techniques as laser particle size analysis, X-ray diffraction (XRD), microscopical observations and microhardness measurements. XRD is certainly one of the most prominent and powerful techniques for microstructural investigation, and is especially useful for materials processed by MA. Although, contrary to transmission electron microscopy (TEM), it does not provide direct images of the microstructure, it has some undeniable features. XRD is reliable statistically, as each intensity peak represents an average over multiple coherently scattering domains of the diffracting substance [4]. TEM measurements, on the other hand, are restricted to the very tiny fraction of the investigated object, so questions may arise if these measurements are representative of the whole sample [5]. Furthermore, in cases of severely deformed materials, of which milled powders are a good example, density of dislocations can cross the threshold ($\rho >\sim 10^{15}/m^{2})$ beyond which they become no longer distinguishable and quantifiable in TEM images [6,7]. XRD is also appreciated mainly for its sensitivity for the atomic arrangement and the effortless sample preparation and the measurement itself. Unfortunately, the observed diffraction profiles are rather complex convolutions of physical and instrumental effects, each of them adding its own contribution to peak shapes [4]. As a consequence, the right separation of these contributions is a major challenge in XRD line profile analysis (LPA). If these problems are overcome, it will provide a relatively simple and efficient technique for determining the microstructural properties of nanomaterials. Traditional LPA is generally carried out using one of the so-called integral breadth (IB) methods [8]—Scherrer and Williamson-Hall procedures or their subsequent modifications.

Further developments in the field of X-ray diffraction led to the alternative approach of line profile synthesis (LPS), involving direct modelling of all peak profiles in the diffraction pattern in terms of physical parameters. So far, the most prominent example of this philosophy is undoubtedly the Rietveld refinement method [9], based on adapting model profiles to the experimental pattern by means of a suitable minimization algorithm. Following this trend, the Whole Powder Pattern Modelling (WPPM) has been proposed as a universal technique for microstructure refinement. It provides detailed data on specimen microstructure by directly comparing model peak profiles with the entire experimental pattern, while also considering instrumental effects and background, without imposing any arbitrary fitting function [4,6]. Size and defect contributions are convoluted together with the instrumental component, and the pattern is then directly synthesized through Fourier transformation [10]. As a result, a set of microstructural parameters can easily be extracted from experimental data. Another approach, very similar to WPPM is known as the Convolutional Multiple Whole Profile fitting (CWMP) [11], with the procedure of employing the instrumental profile being the only major difference. WPPM has been successfully utilized for investigating the microstructural evolution of many ball-milled powders and other nanocrystalline materials [10]. Its algorithm has been implemented in a free and flexible software package, named PM2K [12], which was exploited in this study.

As the final microstructure and mechanical properties of sintered alloy are strongly associated with the powder properties (mainly grain size and dispersoids distributions) obtained after MA, it is certainly a fundamental step in the fabrication of powder metallurgy materials. Therefore, basic issues and properties related to ball milling of the ODS alloy composition, such as powder particle size and evolution of microstructure have to be known in order to optimize the process. Generally, this study is designed to yield nanocrystalline and homogeneous ODS powder mixtures for use in further synthesis by spark plasma sintering (SPS) or other sintering techniques. Hence, three ODS powder mixtures of nominal composition of Fe-12Cr-2W-0.3Y_2_O_3_, with various additions of Zr (0.3, 0.5 and 1.0 wt.%) were processed by MA and their important properties were comprehensively evaluated. Key microstructural parameters, mainly crystallite size and dislocation density, were quantified using the state-of-art XRD LPA and are thoroughly discussed. Similar studies exploiting more advanced methods of extracting physically sound microstructural data, especially in the case of multicomponent alloys with overlapping peak reflections and severe anisotropic line-broadening effects, which was the case in this study, are rather rare. Extensive LPA was conducted to extract valuable insights into the MA, as classical, single-line methods (e.g., Williamson-Hall relation) for separating size and strain broadening effects, although still commonly used in the field, are unreliable and their data is difficult to interpret in terms of microstructure parameters.

## 2. Materials and Methods

### 2.1. X-ray Line Profile Analysis

The XRD data was collected on a laboratory D8 Advance powder diffractometer (Bruker, Karlsruhe, Germany) in Bragg–Brentano focusing geometry, equipped with a Cu radiation source produced at 40 kV/25 mA. The powder samples intended for XRD measurements were collected at specified time intervals during MA and immediately scanned to avoid undesirable oxidation. Each powder pattern was gathered in 25°–125° 2θ range using 5 s acquisition time per 0.01° step size. The instrumental profile component of the diffractometer used in this study was determined by scanning a corundum (Al_2_O_3_) line profile standard (NBS SRM 1976b) with high accuracy in a wide Bragg angle range (20°≤2θ≤155°). NBS SRM 1976b is a known and convenient choice as an instrumental broadening standard with certified lattice parameters. Acquired high-definition experimental reflections (with a total of 44, few peaks were omitted due to their very low intensity) were measured in PM2K (considering Kα_1_/Kα_2_ Cu radiation doublet) and the instrumental-related broadening was parametrized according to the Caglioti et. al. formulae [13], following the procedures widely mentioned in the literature (e.g., [8,14]).

In order to consider strain contribution in WPPM analysis, the so-called relevant average dislocation contrast factor $\bar{C}$ has to be known for studied material. In the case of cubic lattice systems, $\bar{C}$ is a linear function of the fourth-order invariant of the hkl Miller indices of the following peaks [15], as shown in Equations (1) and (2):

(1) $$\bar{C}={\bar{C}}_{h00}\left(1-qH^{2}\right)$$

where

(2) $$H^{2}=\frac{h^{2}k^{2}+h^{2}l^{2}+k^{2}l^{2}}{{\left(h^{2}+k^{2}+l^{2}\right)}^{2}}$$

where $\bar{C}$ is the average dislocation factor, where the average is made over the equally populated equivalent slip systems. ${\bar{C}}_{h00}$ is the average contrast factor corresponding to the h00 reflection, whereas the value of q determines the edge/screw type of dislocations [16]. The value of $\bar{C}$ depends only on the ratios of the material’s elastic constants *c*_11_, *c*_12_ and *c*_44_, which can be reduced to two parameters—elastic anisotropy $A_{i}=2c_{44}/\left(c_{11}-c_{12}\right)$ and the ratio $c_{12}/c_{44}$ [17]. As the elastic properties of alloys compositions presented in this work are unknown, the model proposed by Giri [18] was used to approximate them, according to Equation (3):

(3) $$\frac{a_{0}^{Fe12Cr2W}}{c_{ij}^{Fe12Cr2W}}=\delta^{Fe}\frac{a_{0}^{Fe}}{c_{ij}^{Fe}}+\delta^{Cr}\frac{a_{0}^{Cr}}{c_{ij}^{Cr}}+\delta^{W}\frac{a_{0}^{W}}{c_{ij}^{W}}$$

where Fe, Cr and W superscripts refer to the specific element, the Fe12Cr2W superscript refers to Fe-12Cr-2W alloy, $a_{0}$ is the lattice constant, *δ* is the weight fraction (wt.%) of the element and $c_{ij}$ is the specific elastic constant. The impact of both Y_2_O_3_ and Zr elements on the elastic parameters was neglected because of their very small mass fraction (≤1 wt.%) in the powder mixtures. The $\bar{C}$ values obtained for Fe-12Cr-2W were imposed for all subsequent calculations, regardless of Y_2_O_3_ and Zr content. Single crystal elastic constants of α-Fe [19], Cr [20] and W [21] were adopted from the literature, while their lattice parameters were taken from Bruker’s DIFFRAC.EVA suite database. Lattice constant of alloy was measured by XRD. The $A_{i}$ values illustrate that ferrite anisotropy can be substantially restricted by the addition of Cr and W; their mass fractions are, however, insufficient to change the strain nature of the alloy to isotropic [22].

**Figure 1:**
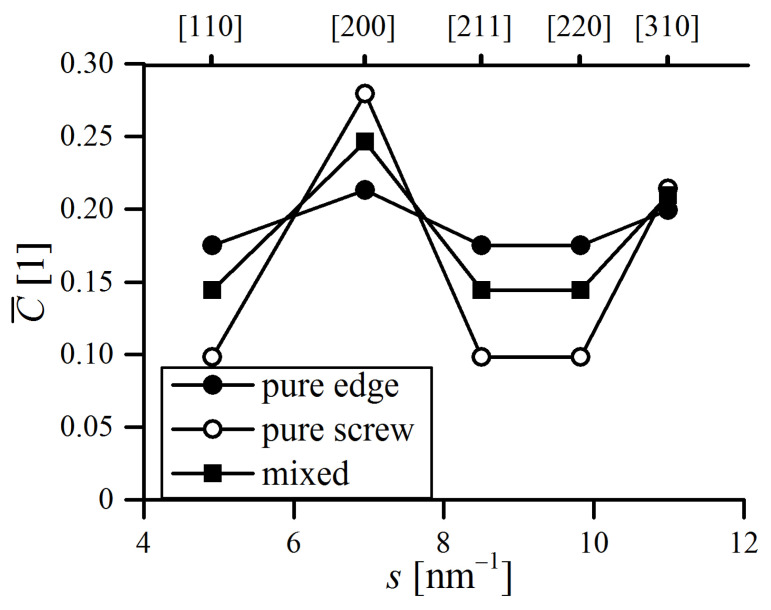
Average dislocation contrast factor trend as a function of diffraction vector, designated for presented alloys. Calculated values are for edge (dot), screw (hollow dot) and mixed 50%–50% edge-screw (square) dislocation character. Miller indices are indicated for each family of equivalent planes.

The data needed to implement the dislocation broadening model are collated in Table 1. The ${\bar{C}}_{h00}$ values for edge and screw (${\bar{C}}_{h00}^{e}$ and ${\bar{C}}_{h00}^{s}$, respectively) dislocations were calculated using the online program Anizc [23], with regards to the primary edge 〈1,1,1〉{1,1,0} and screw 〈1,1,1〉 slip systems for dislocations in bcc metals. Theoretical q values for pure edge and pure screw character ($q^{e}$ and *q_s_*) were determined by equations provided by Ungár et al. [17]. After obtaining ${\bar{C}}_{h00}$ and q, the average contrast factor can be calculated. Trend of $\bar{C}$ as a function of the scattering vector modulus (s=2sinθ/λ) is plotted in Figure 1. As presumed, $\bar{C}$ reaches its maximum towards h00 direction, which is the soft crystallographic direction in Fe-bcc, along which the strain proceeds the most easily [24].

### 2.2. Microscopic Observations of Mechanically Alloyed Powders

Powders at various stages of MA, intended for microscopic observations, were prepared analogous to samples intended for microhardness testing, but additionally etched. They were mounted on a steel roundel using cyanoacrylate adhesive. After the glue had fully cured, they were gently ground using successive fine grades of abrasive papers and finally polished to a mirror finish using polishing pads soaked with extra fine alumina slurry (0.05 μm). Before etching, the samples were washed in warm methanol using ultrasonic cleaner to rinse any possible residue from the abrasive media. The etching reagent used to reveal the underlying microstructure of powders was Kalling’s 2 reagent (also known as waterless Kalling’s), prepared by mixing methanol, hydrochloric acid and copper chloride dihydrate in the ratio of 1:1:0.05. As it turned out, etching of powders after MA was somewhat difficult due to severely deformed microstructure and the anti-corrosive nature of stainless steel, as it required relatively long etching times (up to several minutes), especially for powders milled longer. An Lext OLS 4000 (Olympus, Tokyo, Japan) laser confocal microscope was used to take optical microscopy (OM) micrographs of powders at various time points of MA ($t_{MA}$). SEM observations supported with EDS analyses were carried out using a Scios 2 DualBeam microscope (Thermo Fischer Scientific, Waltham, MA, USA).

### 2.3. Laser Diffraction Size Measurements

The size and distribution of powder particles (both in “as-received” and during MA conditions) were measured using the laser particle sizer ANALYSETTE 22 MicroTec plus (Fritsch, Idar-Oberstein, Germany). All measurements were conducted in wet dispersion units in the range of 0.08–2000 μm. Each powder sample was initially dispersed in water until the proper suspension was formed and measured thrice. The ultrasounds (100 W/36 kHz) were used to aid optimal dispersion and limit the presence of large powder agglomerates, which could significantly disturb the results. Therefore, after a single measurement cycle, particles were additionally subjected to a 10 s sonification interval. The size of particles was calculated on the grounds of Fraunhofer’s optical law.

## 3. Results

### 3.1. Kinematic Characterization of Pulverisette 6 Ball Mill

It is well known that the general outcome and efficiency of MA is strongly dependent on set process parameters. In order to give deeper insight into the basic physics involved in MA and to model the process effectively, kinematic equations describing the balls’ motion will now be presented. Modelling of MA was carried out to settle the salient factors affecting the process and to optimize it for a particular application. However, as the nature of MA is inherently stochastic [25], realistic intensions of MA modelling described herein are restricted to estimate general trends rather than precisely predicting the outcomes.

The kinematic equations describing the ball movement inside the jar and consequent ball-to-powder energy transfer were derived by Burgio et al. [26]. Magini and Iasonna [27] implied that the prevailing energy transfer during milling is via collisions, at least when the filling charge of the vial is relatively low, and this assumption underlies subsequent calculations in this section. Although most researchers agree that collisions are assumed to be the primary energy transfer, incident, sliding, rolling and friction may also be relevant phenomena [28]. Nevertheless, the kinematic and energetic relationships of the MA system used herein were designated on the basis of the Magini–Iasonna collision model, that is widely described in literature (e.g., [26,27,29]).

**Figure 2:**
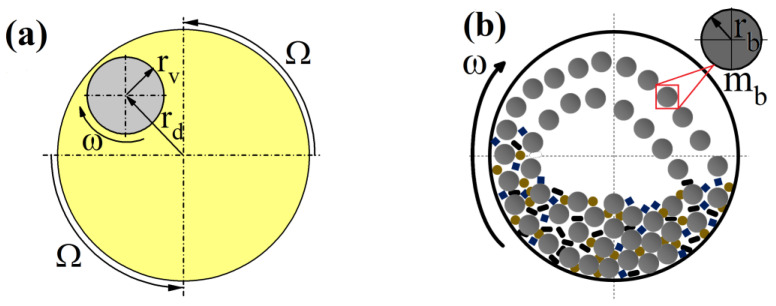
Schematic representation of movement and dimensions of Pulverisette 6 (**a**) and balls inside milling vial (**b**).

The geometrical dimensions and dependencies of the Pulverisette 6 (Fritsch, Idar-Oberstein, Germany) milling equipment are visualized in Figure 2 and summarized in Table 2. Accordingly, $r_{d}$, $r_{v}$ and $r_{b}$ are the main disc, vial and ball radii, respectively, h_v_ is the height of the jar, $m_{b}$ is a ball’s mass and i=ω/Ω is the ratio of vial’s rotational velocity (ω has a negative value as it turns in opposite direction than main disc) with respect to main disc’s angular velocity (Ω).

**Figure 3:**
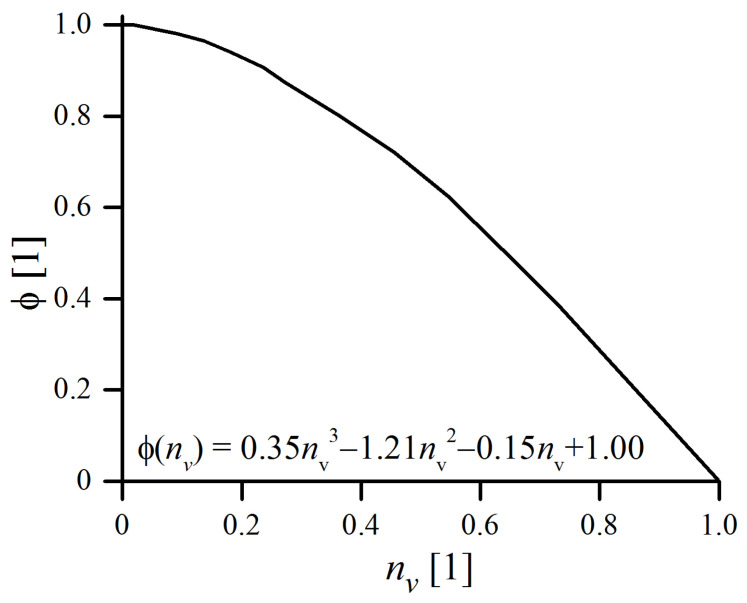
Yield coefficient versus degree of the vial’s filling $\phi (n_{v})$, in the case of using ø10 mm balls (calculated after [26]).

The energy transfer fundamental to the MA process is the one from the balls to the powder, which is only a fraction of the total energy output to the system. Therefore, it would certainly be more useful to specify the energy transfer going only to the powder, which can be calculated as a ratio of impact energy of balls $E_{b}$ to the maximum quantity of material trapped between balls Q, approximated by the powder adhering to the ball surface. Detailed derivation of the proper formulas can be followed in the literature ([27,29,30]), so it will only be mentioned here briefly. After substituting $m_{b}=\left(4/3\right)\pi r_{b}^{3}\rho_{b}$, where $\rho_{b}$ is the density of milling balls, the equation considering energy input to the powder takes the form:

(4) $$E_{b}/Q=0.1532\phi r_{b}\rho_{b}^{\frac{3}{5}}v_{b}^{\frac{6}{5}}E^{\frac{2}{5}}/\psi$$

where ϕ is the function related to the degree of filling of the jar, $v_{b}$ is the relative impact velocity of a ball and *ψ* is the surface density of the powder covering the balls. The ϕ value with respect to the filling factor $n_{v}$ is plotted in Figure 3. It is equal to 0 in the case where the vial is completely full (no possible movement of balls) and ϕ=1 when the vial is nearly empty. When the degree of filling becomes greater, reciprocal collisions are no longer negligible, as the collisions themselves become less productive due to shorter free paths available for ball and, consequently, lower relative impact velocities [29].

The ball impact velocity $v_{b}$ depends on kinetic (ω/Ω) and geometrical (*r_d_*, $r_{v}$ and $r_{b}$) relationships (Table 2). Therefore, for each rotation speed of the main disc (Ω), the relative impact velocity can be simply obtained using the Equation (5):

(5) $$v_{b}=K_{b}\Omega$$

For Pulverisette 6, after substituting the geometrical constants (Table 2), and when using ø10 mm balls, $K_{b}$ = 0.1658. It should be underlined that the value of $K_{b}$ is valid only for the discussed MA setup, and would be different when changing the process conditions (e.g., different milling device or balls’ size).

Finally, after inserting the material ($E=2.1\times 10^{11}$ Pa, $\rho_{b}=7640$ kg/m^3^), and dimensional ($r_{b}$) constants for stainless steel, and using the previously derived relation that $v_{b}=K_{b}\Omega$ (Equation (6)), the following formula is obtained:

(6) $$E_{b}/Q=640.4\phi \Omega^{\frac{6}{5}}/\psi$$

As can be noticed, energy absorption of the powder for can be easily obtained using Equation (6) for the given rotational speed Ω and the vial’s filling degree ϕ.

**Figure 4:**
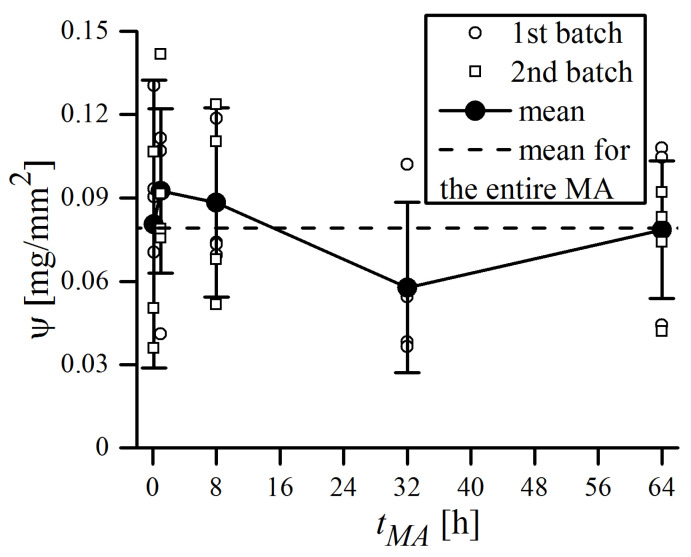
Surface density of the powder covering balls. Hollow circles and squares distinguish between the measurements made for two different batches. Filled circles represent the mean value of all measurements (two batches) for a given $t_{MA}$, while dashed lines are the mean value of all measurements over the entire MA process. Error bars represent standard deviation of the measurement.

The crucial point in Equation (6) concerns the amount of powder subjected to each impact, which is assumed to be equal to that adhering to the balls’ surface, i.e., the surface density of the covering powder ψ. It is realistic to assume that the ψ value should represent the minimum quantity of trapped material, as some powder, not necessarily adhered to the balls, may also be involved in the collision event. Its value is probably dependent on many circumstances—the BPR, type and initial size of the powders used, type of balls, etc., and thus, is unreliable to presume arbitrarily. Moreover, the value of ψ certainly varies during the MA process, as most powders are more adherent during the early stages of the process, when the particles are relatively soft and have high affinity to weld together (welding predominance over fracturing) [5]. Therefore, ψ is apparently the major source of uncertainty in Equation (6), and should be obtained from direct weight measurements for the individual MA process. In this study, a few balls were carefully taken out from the vial after the specified MA time, each of them was placed in separate ceramic crucibles, and weighed altogether using a precise balance (Mettler Toledo XS205). Then, the balls and crucibles were cleaned of powder residues and weighted again. The resulting difference, i.e., the mass of the adhering powder, related to the ball surface area was defined as ψ. The *ψ* values measured during MA are plotted in Figure 4.

**Figure 5:**
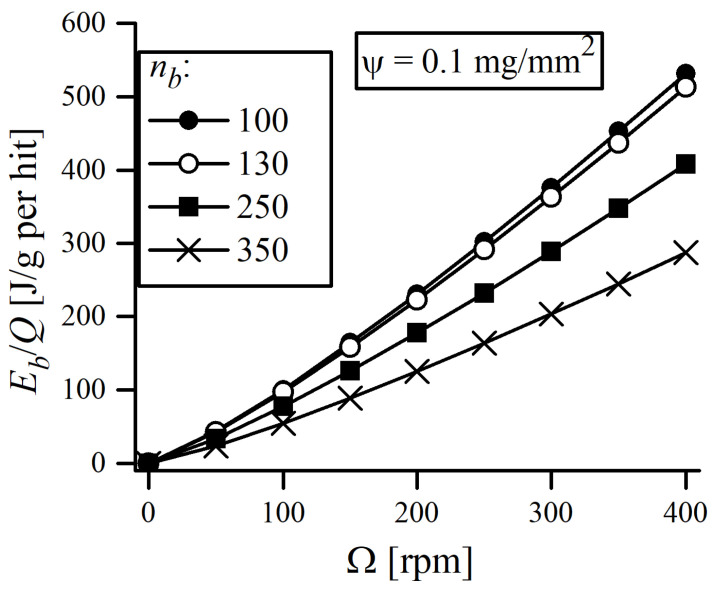
Energy transferred per unit mass of powder per single hit as a function of the mill rotational speed. Data plots were created for various number of balls $n_{b}$ (different filling level of jar) and assuming a constant value of ψ = 0.1 mg/mm^2^, independent of the quantity of balls used.

As can be seen, the mean ψ value averaged over the entire MA time is 0.079(25) mg/mm^2^, and none of the single measurements ever exceeded 0.15 mg/mm^2^. This is less than the typical values of ψ that can be found in the literature. Magini et al. [27] reported a mean ψ = 0.3 mg/mm^2^ for the Pd-Si system milled in Pulverisette 5, whereas Suryanarayana [5] claimed that around 0.2 mg of powder is trapped during each collision (without reference to the surface area). The average measured value of ψ should constitute a minimum of the real quantity of material involved in single collision event, as the additional amount of powder, not necessarily adhering to the surface, may also be trapped between balls. This, plus the fact that single measurements, conducted even at the same batch and time of MA, may actually vary by a factor of ~4, makes ψ the primary source of uncertainty in the presented calculations. Therefore, it highlights the importance of measuring ψ for each milling system and type of powders used, as large discrepancies may arise when the literature values are assumed arbitrarily. The results of the energy transfer calculations are reported in Figure 5, in form of an “energy map”, which evaluates the energy transferred per unit of powder mass.

The energy map can be used to estimate the required amount of energy to be transferred into the powder to obtain a solid solution of ODS alloy. According to Figure 5, the most straightforward method for increasing the energy transfer to the powder is by simply increasing the rotational speed Ω. This is, however, accompanied with a rise in temperature inside the milling jar. In extreme cases, it may lead to the overheating the powder, causing its degradation and welding with the balls. Here, the rotational speed of 300 rpm was found to be the upper safe limit of Ω, as increasing the Ω to 350 rpm caused welding of particles to the balls. Although it is difficult to estimate the maximum local temperature (at the site of collision) inside vial, the temperature of the outer surface of the jar, measured by a pyrometer, was found to be 56 ± 4 °C when Ω = 300 rpm.

The energy map can be used to estimate the balance between maximizing the energy of impacting balls and the net yield of a MA batch. In this study, a yield of ~50 g of powder in a single batch was required for further processing and sintering by the spark plasma sintering (SPS) method. This, while preserving the typical 10:1 ball-to-powder ratio (BPR), required the use of 130 balls. As can be noticed, using 130 Ø10 mm balls had a negligible impact on energy transfer. The principal conditions of MA used in this study are presented in Table 3. The process is usually performed in a protective atmosphere to prevent excessive oxidation of the batch. Ar can be perceived as a standard inert atmosphere and has been widely used, although some researchers found using He or H_2_ to be more beneficial [31,32]. An important incentive to use a H_2_ atmosphere is a lower oxidation degree of milled powders [31]. During MA, H_2_ can react with metal oxides (MO) and reduce them, accordingly to $MO+H_{2}=M+H_{2}O$, which may be a reasonable explanation of O reduction in powders milled in a H_2_ atmosphere. As a consequence of elevated temperature inside the milling jar, the H_2_O can turn into steam and further react with the C impurities contained in the jar: $C+H_{2}O_{\left(steam\right)}=CO+H_{2}$. CO can also react with MO: $MO+CO=M+CO_{2}$. Gaseous byproducts of the redox reaction, CO and CO_2_, can be easily removed from the jar during purging. Bearing in mind the above statements, we decided to use H_2_ as a protective MA atmosphere for the purposes of this work.

### 3.2. Characterization of Powders Prior to Mechanical Alloying

One of the biggest challenges of developing ODS steels for fusion applications is that the elements used in the alloy must fulfill certain criteria, so only a few of them can be considered. Most importantly, in case of ferritic (FS) and ferritic–martensitic (F/M) steels, these elements must form or stabilize the bcc structure and meet the low-activation requirement, ensuring that radioactivity from the material decays to low levels in less than 100 years. For the purpose of this study, ODS RAF alloys were manufactured using the commercially available, high purity Fe, Cr, W and Zr metallic elemental powders and Y_2_O_3_ nanoparticles. A brief description of the characteristics of the initial powders are listed in Table 4.

**Figure 6:**
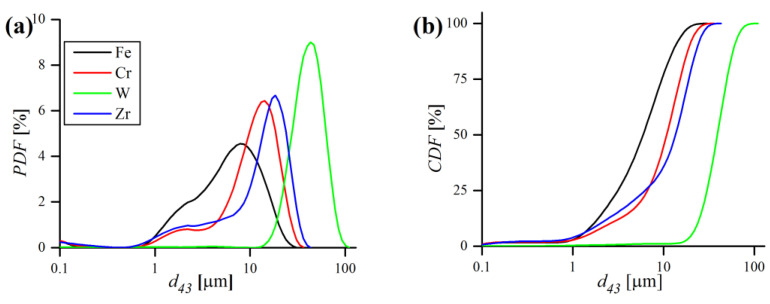
Probability (**a**) and cumulative (**b**) density distribution functions of elemental metallic powders.

The substantial properties (size and morphology) of the initial powders prior to milling were determined by laser particle size measurements, XRD and SEM observations. Particle size measurements of starting elemental powders, in terms of probability (PDF) and cumulative density distribution functions (CDF) are presented in Figure 6 and listed in Table 4.

**Figure 7:**
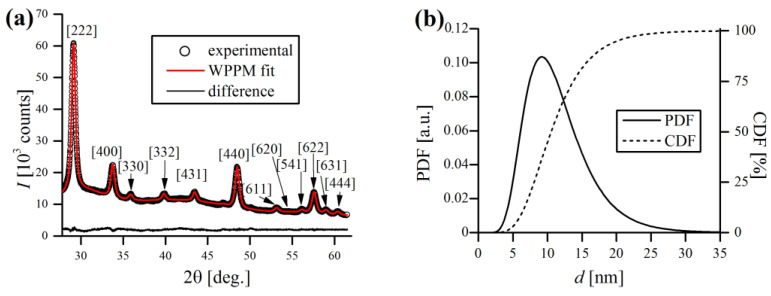
XRD pattern of Y_2_O_3_ powder used in this study with WPPM fit (**a**) and corresponding crystallite size distribution (**b**).

According to the results, Fe, Cr and Zr powders are fine and have a similar $d_{43}$ of ~10 µm, while W powder is considerably larger (~40 µm). The W powder size distribution is unimodal and symmetrical, whereas other powders PDFs are rather bimodal, with one major peak and a less pronounced minor one, in the very fine size range (Figure 6a). The size of Y_2_O_3_ particles could not be measured on laser analyzer, as their size is smaller than minimal admissible size (0.08 µm) for this device. Therefore, the crystallite size of Y_2_O_3_ was calculated by the WPPM algorithm. Figure 7a shows the diffractogram of pure Y_2_O_3_ with WPPM fit with lognormal domain size distribution (Figure 7b). XRD revealed that used yttria has a cubic, corundum-like crystal system (Ia$\bar{3}$ space group), with a lattice parameter $a_{0}$ = 10.6148(2) Å. As can be noticed in Figure 7a, the Y_2_O_3_ peaks are rather broad, even before the mechanical treatment during MA, which indicates very small crystallite sizes. Indeed, the WPPM calculations confirmed the small mean domain size of Y_2_O_3_ of d = 11.5(4.7) nm (Figure 7b), without any detectable strain broadening effects (ρ~0).

**Figure 8:**
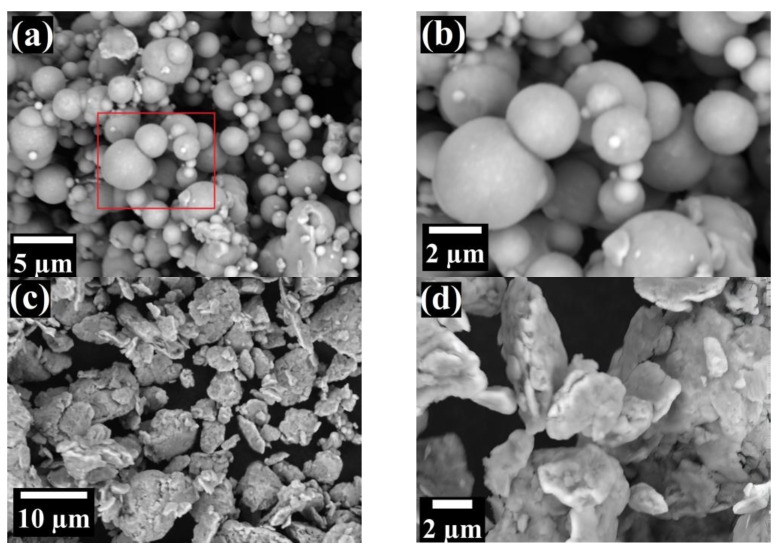

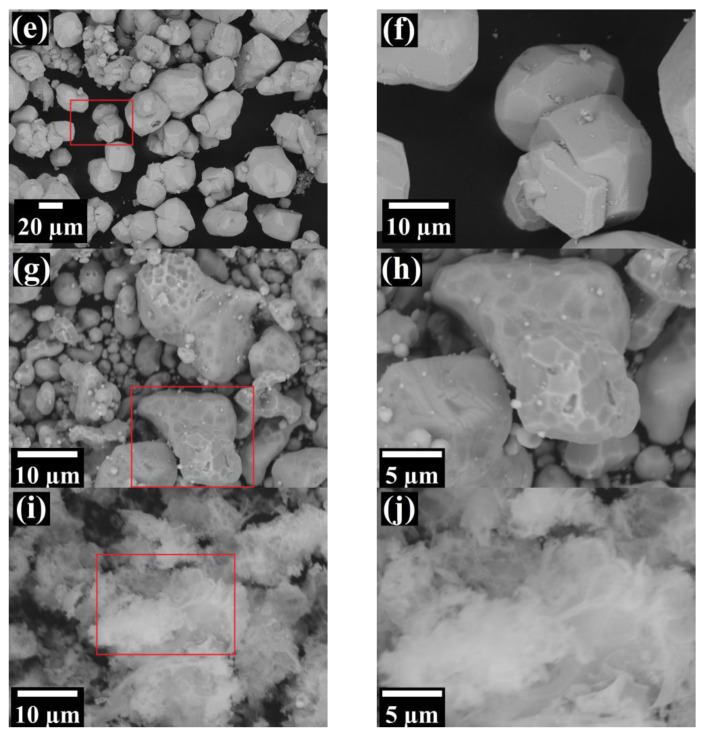
SEM micrographs of elemental powders utilized in this study—Fe (**a**,**b**), Cr (**c**,**d**), W (**e**,**f**), Zr (**g**,**h**), Y_2_O_3_ (**i**,**j**). Red square marks the area being magnified in the next photo.

SEM investigation of the elemental powders revealed substantial differences in their shape. Even after a brief look, Fe particles are definitely the finest and are almost ideally spherical, having smooth, featureless lateral surfaces (Figure 8a,b). The main fraction of Fe globes of ~5 μm diameter were surrounded with very fine (<1 μm) satellite spheres. Contrarily, Cr particles were considerably larger, flat and flaky, with rather rounded edges (Figure 8c,d). W powders were spheroidal with multiple visible faces, which are similar to the shape of a polyhedron (Figure 8e,f). Zr particles were spheroidal and have rounded edges, similar to pebble stones (Figure 8g,h). Moreover, their lateral surfaces were clearly decorated with shallow, superficial holes. The particles of Y_2_O_3_ appeared on SEM micrographs as dendritic, feather-like structures with irregular, jagged boundaries (Figure 8i,j).

**Figure 9:**
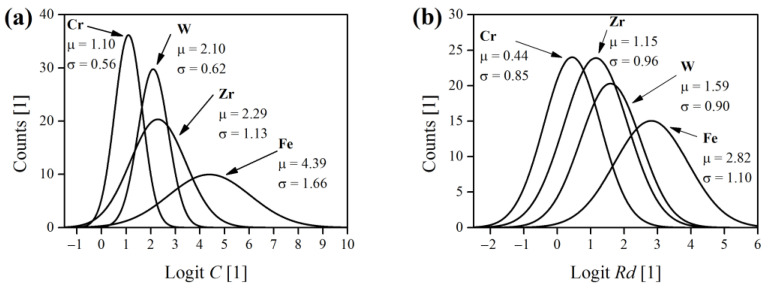
Normal distributions of circularity C (**a**) and roundness Rd (**b**) for elemental metallic powders. μ and σ are normal mean and standard deviation, respectively.

Besides visual examination, more detailed image analysis was carried out using ImageJ software to mathematically determine the shape of the powders. Circularity C and roundness Rd are common shape indices used to characterize the grain form. In ImageJ, C and Rd are calculated according to the Cox [33] and Pentland [34] equations, respectively. Circularity is defined as the ratio of area to perimeter, with unity indicating a perfect circle. Lower values of C are characteristic for irregular and angular shapes. Similarly, roundness distinguishes particles with circular cross-sections from less circular ones (elliptical, etc.). As the values of dimensionless shape descriptors are in the range from naught to unity, the data was transformed using the logit function for the statistical analysis of data, using the following relation (Equation (7)) [35]:

(7) Logit(S)=ln[S/(1−S)]

where S is the shape descriptor (herein C or Rd). Distribution of logit-transformed shape indicators was plotted as a histogram, using a bin size of 0.5, and was estimated using the normal distribution function. The obtained normal curves are demonstrated in Figure 9.

It can be stated that the shape of Fe powders was most similar to a regular sphere, considering that the normal, logit-transformed mean values of C and Rd were the highest among the other powders (Figure 9). On the other hand, Cr grains were most angular and irregular, and also deviated the most from the shape of a circle, with W and Zr powder shapes being somewhere between Fe and Cr extremes.

### 3.3. Microscopic Observations of Mechanically Alloyed Powder

**Figure 10:**
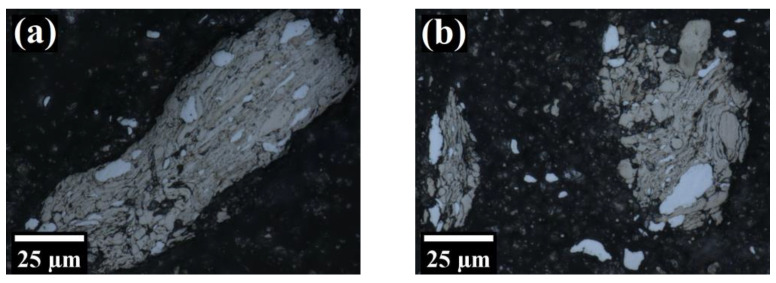

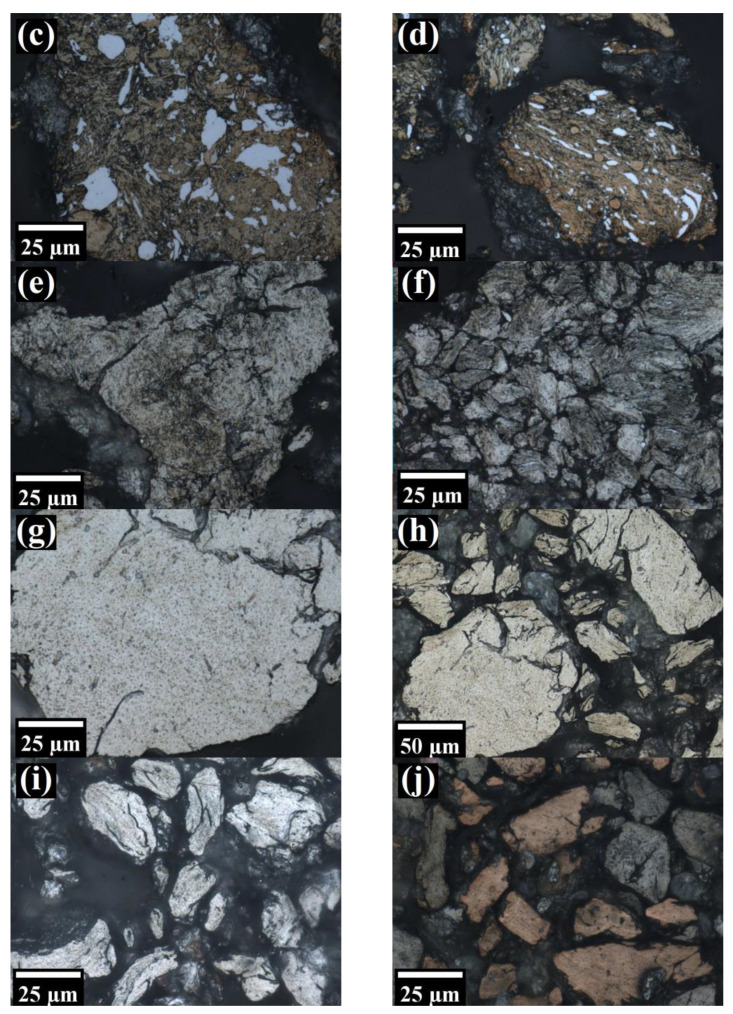
Laser confocal microscope micrographs of Fe-12Cr-2W-0.5Zr-0.3Y_2_O_3_ powder alloyed for 1/6 h (**a**,**b**); 1 h (**c**,**d**); 8 h (**e**,**f**); 24 h (**g**,**h**); 64 h (**i**,**j**).

As depicted in Figure 10a,d, at early MA stages the particles form conglomerates consisting of multiple finer, elongated particles with clearly visible boundaries separating them from each other. Because the starting powders are relatively soft (proven later by microhardness measurements), the flattened layers tended to overlap and form cold welds. At short $t_{MA}$, this lead to the formation of layered composite particles, consisting of a mixture of starting ingredients, which was recognized as a lamellar structure. In addition, the distinctive white sub-particles were clearly distinguished in these micrographs. As confirmed later by SEM EDS observations, these are W particles, which are not susceptible to etching by the reagent used.

As the MA process proceeds, the work hardening becomes more pronounced and the amount of defects introduced to the crystals seriously increased, providing short-circuit diffusion paths and facilitating the formation of an alloy [5]. Moreover, the particles became harder and more susceptible to fracture. As shown in Figure 10e,f, after 8 h of MA the individual constituents of lamellae were fine and hardly recognizable due to fragmentation of the fragile flakes. At this stage, the particles consisted of convoluted lamellae, a result of microstructure refinement and interdiffusion of the constituents.

At later stages of MA, the lamellae became even finer and more convoluted and no longer resolvable under the optical microscope (Figure 10g,j). Eventually, a refined and homogenized microstructure was attained, with the absence of any conspicuous microstructural features. It should be noted that it is difficult to unambiguously estimate the structural features (e.g., grain size) of powders after MA due to lack of clarity in OM observations in such complicated microstructures. At the last stage of MA, featureless micrographs suggested a nano-sized microstructure of the powder.

**Figure 11:**
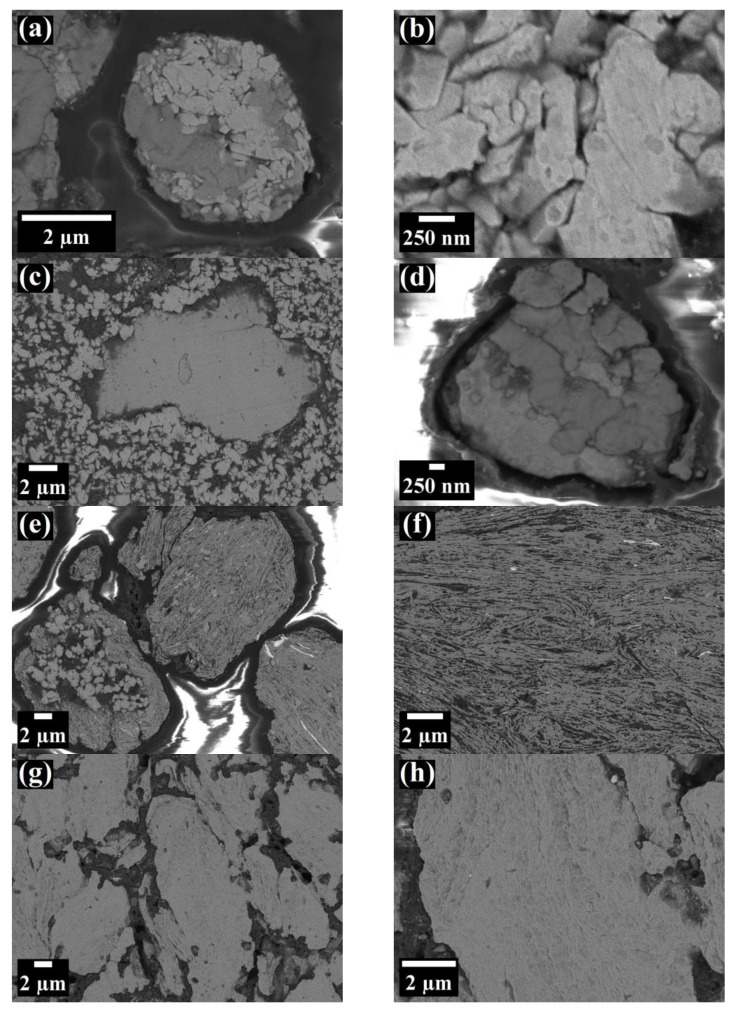
SEM micrographs of etched surface of Fe-12Cr-2W-0.3Zr-0.3Y_2_O_3_ ODS steel powders after 1/6 h (**a**), 1/2 h (**b**), 8 h (**c**) and 64 h (**d**) of MA.

In order to perform a more comprehensive analysis, subsequent SEM observations of etched cross-sections of the mechanically alloyed ODS powders were carried out. The results of these observations are presented in Figure 11. Similar conclusions to the OM observations can be drawn in the case of short MA time—the particles were in form of agglomerates consisting of loosely bound sub-particles, with clearly visible boundaries (Figure 11a–d). In addition, large, loose, single W particles, surrounded by much finer particles of the other chemical can be discerned (Figure 11c). Despite the short time of the process, very fine (few hundred nm) sub-grains can be already observed at high magnification (Figure 11d).

At the intermediate phase of MA (8 h), SEM revealed a very specific, fibrous microstructure of the alloyed powders (Figure 11e,f). Some of the particles showed a mixed microstructure, consisting of fine (<1 µm), rather round, sub-particles in the interior and uniformly layered structure near the outer surface area (Figure 11e). Characteristic, white and elongated W sub-particles, although much finer, could still be observed inside the grains.

The powders after MA (64 h) presented uniform microstructures without inhomogeneous inclusion or other characteristic features (Figure 11g,h), suggesting that chemical composition of the individual particles approached that of the overall, nominal composition of the starting blend. Therefore, it indicated that alloy formation was finished. Additionally, at higher magnification, fine and elongated shapes, possibly representing sub-grains, can be distinguished (Figure 11h). Generally, very similar SEM micrographs of ODS steel powders during MA were also obtained by other authors in [36].

**Figure 12:**
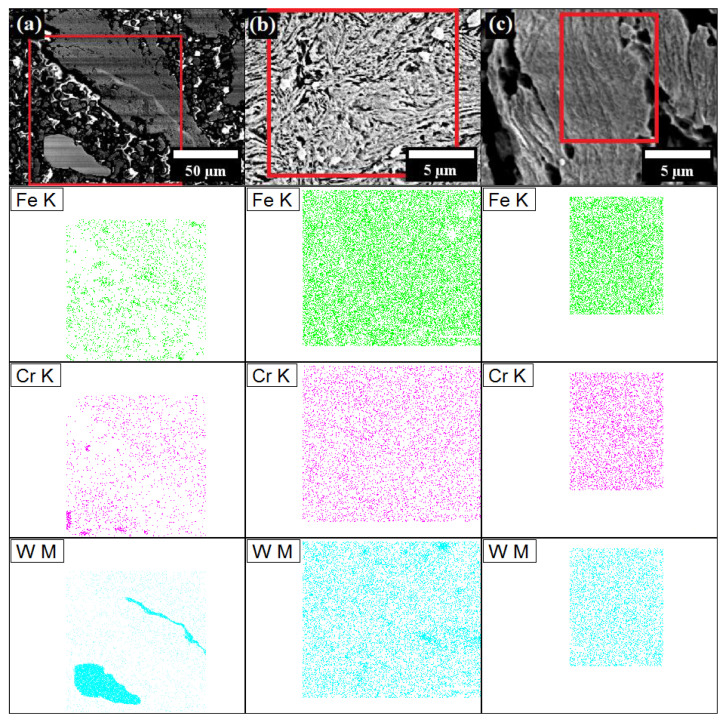
SEM EDS mapping of the selected area of Fe-12Cr-2W-0.3Zr-0.3Y_2_O_3_ ODS powders after 1/6 h (**a**), 8 h (**b**) and 64 h (**c**) of MA. EDS maps of each powder are presented vertically, below the corresponding SEM image on top.

The results of SEM energy-dispersive X-ray spectroscopy (EDS), in terms of chemical mapping of Fe, Cr and W elements is presented in Figure 12. Zr and Y_2_O_3_ were not detected by EDS, possibly mainly due to their very small mass fraction. Other theories to explain this phenomenon can be proposed as follows: first, Zr is added as a small fraction and, thus, is very rapidly dissolved in Fe, which was also confirmed by XRD. Second, nanometric Y_2_O_3_ particles are so small that are below the limit of detection by the EDS detector.

As expected, the results demonstrated that Fe, Cr and W elements are clearly segregated after a short MA time (Figure 12a). In addition, it proved that the larger, white particles are W. After 8 h of MA, the chemical composition of the particles was much more uniform, although, the larger aggregates of W, however, were still detected (Figure 12b). After 64 h, the chemical maps were entirely stochastic, without any larger agglomerates of any element, consequently manifesting that proper solid solution of the elements was achieved (Figure 12c).

### 3.4. Particle Size Evolution during Mechanical Alloying

**Figure 13:**
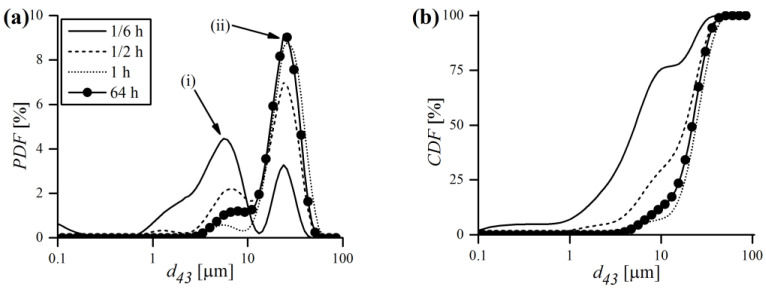
Laser diffraction analysis results of particle size evolution during MA, in terms of probability (**a**) and cumulative (**b**) density functions of Fe-12Cr-2W-0.3Zr-0.3Y_2_O_3_ powder.

Particle size evolution during MA, in terms of probability and cumulative density functions, was obtained by a laser particle sizer and is shown in Figure 13. As can be noticed, the probability distribution is bimodal, with two separate peaks labeled as (i) and (ii) (Figure 13a). At the very beginning of MA (1/6 h), the finer (i) fraction prevailed over the coarser one (ii), and the vast majority (around 75%) of volumetric particle diameters ($d_{43}$) was in the 0.1–10 μm range (Figure 13b). However, just after 1/2 h of milling, most of the (i) fraction decayed, which is accompanied with a mutual increase in the coarser (ii) fraction. It was also manifested by the shift of the cumulative curve towards higher $d_{43}$ values. After 1 h of MA, the (i) fraction almost completely disappeared and the PDF became close to unimodal. At the end of MA, a recurrence of the smaller fraction was observed, manifested by a small rise of the (i) peak.

**Figure 14:**
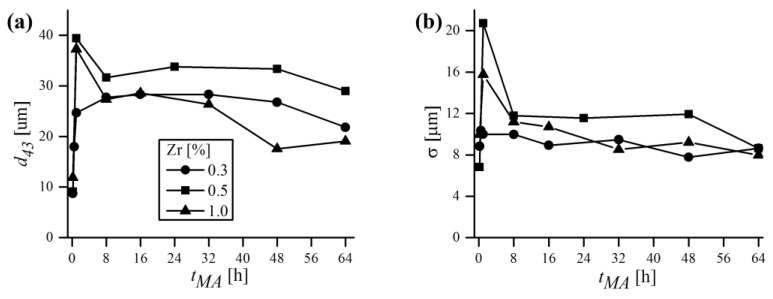
Laser diffraction analysis results of particle size evolution during MA, in terms of mean volumetric diameter (**a**) and standard deviation of distribution (**b**) in function of MA time.

To better visualize the evolution of powder diameters during MA, the mean values of $d_{43}$ and corresponding standard deviation σ were plotted in Figure 14 for three different chemical compositions. Generally, the trends of $d_{43}$ and σ were closely related and mimicked each other. At the beginning, the powders were very small ($d_{43}$~10 μm) and had a rather narrow distribution (σ~8 μm). After 1 h of MA the $d_{43}$ rose exceedingly, with an accompanying growth of σ. As mentioned earlier, in the early stages of milling, particles were relatively soft and therefore more prone to weld together and form bigger particles. As a consequence, a broad range of particles developed, mostly larger than the starting ones, with some of them having a several times larger diameter. With continued deformation, the particles became so strongly work-hardened that they began to break into smaller pieces, which is manifested in Figure 14 by the drop of $d_{43}$ and σ to lower values. It is worth noting, however, that even if at some MA stages the size of particles continued to be the same, their microstructure was still refined due to continued impact from the milling balls, which was supported by XRD. As the process continued, an equilibrium between the rate of welding (increasing the size) and fracturing (refining the size) was attained, and only slight deviations of $d_{43}$ and σ were observed. Finally, alloyed powder batches were obtained, characterized by fine size ($d_{43}$ = 21.8; 29.0; 19.1 μm) and narrow distribution (σ = 8.6; 8.6; 8.0 μm) in the case of 0.3, 0.5 and 1% Zr content, respectively. It is somehow puzzling that the final mean size of particles was higher than the starting one, despite relatively long milling times. Usually, especially in case of using single component or prealloyed powder, the final size after MA is lower than the starting size (e.g., [37]). Herein, the evaluation of this phenomenon is more complicated because the chemical composition consists of multiple starting constituents, varying in size and shape. Therefore, it is plausible that the equilibrium particle size after MA of the powder mixture consisting of finer (Fe, Cr and Y_2_O_3_ in this case) and much coarser (W and Zr) starting powders can be higher than without MA, especially when the initial size is already very fine (~10 μm in this study). Another explanation might be the fact that during MA the powder is constantly contaminated with the material eroded from the vial and balls, which might be coarser than the batch and increase the particle size as a consequence. It is also worth noting that $d_{43}$ may vary significantly even from batch to batch (difference in chemical composition is rather negligible to size evolution) despite using the same equipment and milling conditions, as demonstrated in Figure 14.

### 3.5. X-ray Diffraction and Line Profile Analysis of Mechanically Alloyed Powders

**Figure 15:**
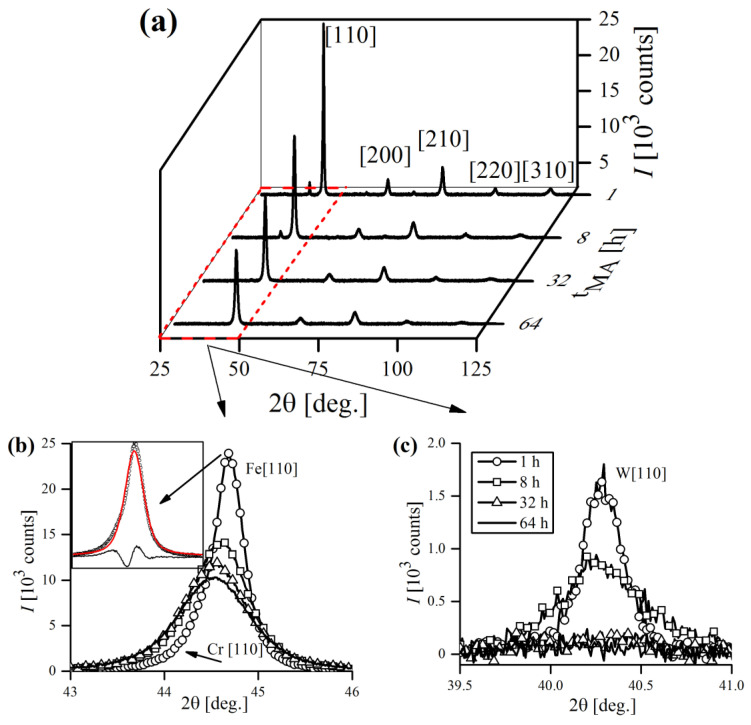
General XRD spectra acquired at different stages of MA (**a**). Major Fe-bcc [110] (**b**) and W-bcc [110] (**c**) reflections were magnified to demonstrate their broadening and shift in 2θ positions as MA process proceeds. Inset in (**b**) shows discrepancies in fitting Fe-bcc [110] peak after 1 h of MA with pseudo-Voigt curve, caused by Cr-bcc [110] presence.

Exemplary, general XRD patterns of the Fe-12Cr-2W-0.5Zr-0.3Y_2_O_3_ powder, taken at various $t_{MA}$ are presented in Figure 15. As expected, the microstructure of an ODS alloy was purely bcc from the beginning to the end of MA, which was proven by five major ferritic peaks appearing in the graph, sequentially indexed with Miller indices (Figure 15a). Cr-bcc phase peaks were also evident, but not so obvious due to severe overlapping with Fe-bcc profiles. As a consequence, they were distinguishable only at the initial stages of MA, until Fe-bcc peaks became sufficiently broad, masking their presence. Figure 15b,c presents the evolution of the most prominent Fe-bcc [110] and W-bcc [110] during the MA cycle. The inset in Figure 15b focuses on Fe-bcc [110] reflection aberrated by the presence of overlapping Cr-bcc [110] peak, that is not obviously visible at first sight. However, the poor quality of the pseudo-Voigt fit clearly revealed the Cr-bcc contribution to the line profile.

Clearly, as the time of MA increased, the reflections were becoming broader, which was also accompanied with a drop in their intensity. The influence of mechanical treatment on line profiles was evident, as well as the anisotropic nature of the broadening (Figure 15b,c). The origins of line broadening could be numerous, but certainly the most influential are size and microstrain factors [38]. Besides broadening, the peaks also exhibited a shift towards lower 2θ Bragg positions, suggesting an increase in lattice cell dimensions, which is consistent with Fe forming a solid solution with alloying additives. The deviation of XRD peak positions during MA has already been observed in analogous studies of ball-milled metals and is attributed to mechanisms related to severe plastic deformation [39].

In general, classical MA is usually a lengthy process, as it requires dozens or even hundreds of hours to complete, depending on the alloy composition and equipment used. The situation is furtherly complicated by a multitude of factors related to MA—type of powders used, rotational speed, type of balls, etc., which most often differ from one study to another, making it impossible to impose a universal milling time. Thus, the process has to be controlled by XRD, and is usually considered finished when all of the alloying additives peaks disappear and only the reflections from matrix phase are left (Fe-bcc in this case). In this study, the MA was regarded as completed when the W [110] peak finally disappeared, which required 56–64 h. The intensity of W [110] reflection was greatly reduced after 32 h; it is still detectable, however, up until 56–64 h of MA (Figure 15c).

**Figure 16:**
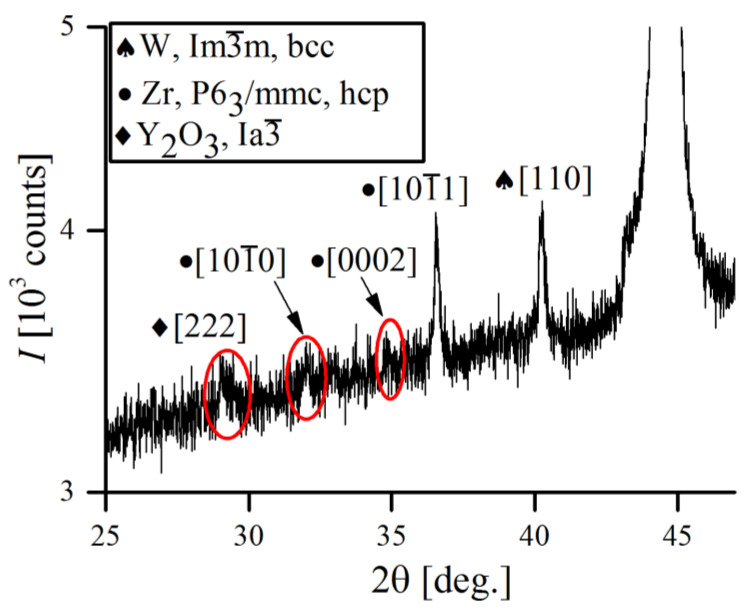
Partial XRD pattern of Fe-12Cr-2W-1Zr-0.3Y_2_O_3_ powder, obtained after initial mixing, proving existence of Zr and Y_2_O_3_ peripheral peaks.

Besides those of the Fe-, Cr- and W-bcc phases, several reflections of minor alloying additives (Zr and Y_2_O_3_) were also detected. However, due to their small percentage in overall alloy composition (≤1 wt.%), they were only detectable at the very beginning of the MA and rapidly vanished just after 1 h of processing, despite the solubility of Y_2_O_3_ in Fe being generally very low [40]. Recent explanations state that severe fragmentation of Y_2_O_3_ particles drives them into amorphous sub-particles, making them undetectable to XRD [41,42]. Other authors also found that homogenization of Y_2_O_3_ in ODS steel during MA was achieved quickly, whereas for metallic components it took much more time [43]. Figure 16 shows the XRD pattern of the low intensity Zr and Y_2_O_3_ reflections obtained after initial mixing of powders (1 min of MA).

**Figure 17:**
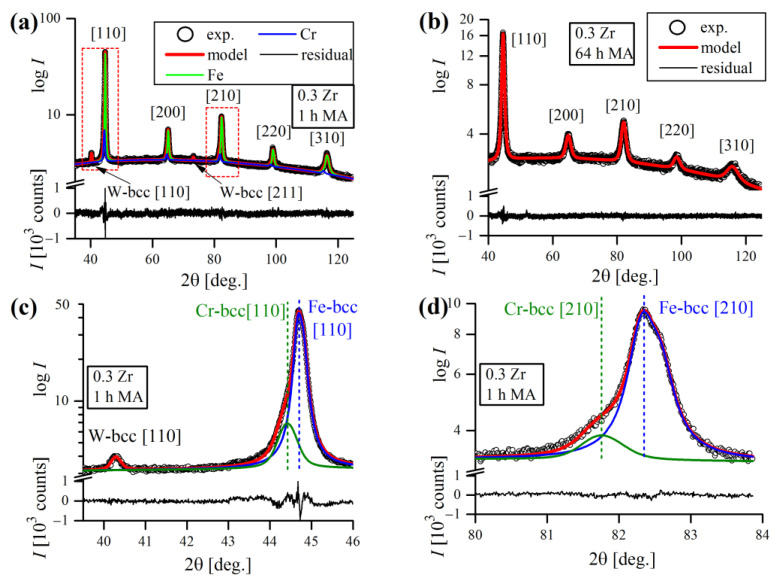
XRD patterns after 10 min (**a**) and 64 h (**b**) of MA refined using WPPM. Modelling details of overlapping Fe and Cr [110], [210] reflections at initial MA stages are presented in (**c**,**d**), respectively.

The quality of WPPM fit can be appreciated in Figure 17, in terms of exemplary refined patterns of Fe-12Cr-W-0.3Zr-0.3Y_2_O_3_ after 1 h (Figure 17a) and 64 h (Figure 17b) of MA. A flat, almost featureless residual line (disagreement between experimental and modelled data) proves good quality of fit. Graphical analysis is generally the primary way to determine the quality of fit and to ensure that the model is correct and chemically plausible, which is particularly important in the case of multiphase patterns with overlapping reflections (Figure 17c,d).

**Figure 18:**
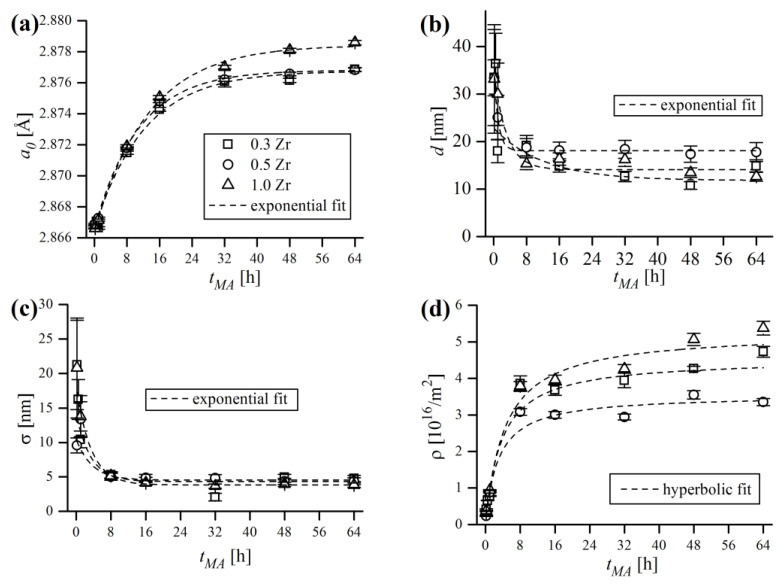
Principal WPPM results of mechanically alloyed powder compositions, in terms of bcc lattice parameter (**a**), lognormal mean domain size (**b**), lognormal standard deviation (**c**) and dislocation density (**d**).

The principal WPPM results of mechanically alloyed powder compositions, in terms of bcc lattice parameter ($a_{0}$), lognormal mean domain size (d), lognormal standard deviation (σ) and dislocation density (ρ) are revealed in Figure 18. It is a common knowledge that MA greatly induces the expansion of the lattice cell due to the combined effects of severe plastic deformation and constant dissolution of alloying additives (Cr, W, Zr, Y_2_O_3_) and, also, due to various contaminants from the milling media into the ferritic matrix during mechanical treatment [6,44]. Volume expansion is further enhanced by fine crystallite size, which increases the solubility of vacancies and other crystal defects [45]. Figure 18a shows the increase of the ferritic lattice constant $a_{0}$, which was particularly sharp at the early stage of MA (up to 16 h) and tended to saturate after 32 h of processing. The data points are almost identical in the cases of 0.3% and 0.5% Zr content, and correspond to 1.06% and 1.04% of the unit cell volume expansion $V_{0}$, respectively (for cubic lattice $V_{0}=a_{0}^{3}$). The mixture containing 1.0% Zr exhibited a slightly larger increase in $a_{0}$, corresponding to a 1.26% rise in cell volume, which is most probably caused by higher Zr content dissolved in the matrix, causing additional expansion of $a_{0}$.

**Figure 19:**
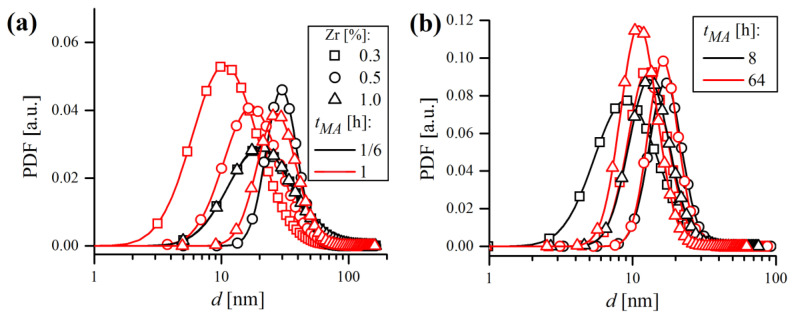
Lognormal distributions of domain size after short (**a**) and long (**b**) milling times.

The analysis of domain size trend (Figure 18b) points out that the reduction of d is especially effective only at initial MA stages (up to 8 h), with only slight reductions being achieved afterwards. Lognormal standard deviation σ (Figure 18c) mimicked the trend of d, which showed that MA not only caused the reduction of mean domain size, but also caused the d distribution to narrow with the milling time. Lognormal distributions of domain size, in terms of probability density functions (PDF), are shown in Figure 19. Grain size distributions are roughly coincident in all samples at a given *t_MA_*, regardless of the Zr content. It can be stated that during MA, the microstructure evolved towards a nanocrystalline state consisting of very fine (d~15 nm), narrowly dispersed (σ~5 nm) crystalline domains.

**Figure 20:**
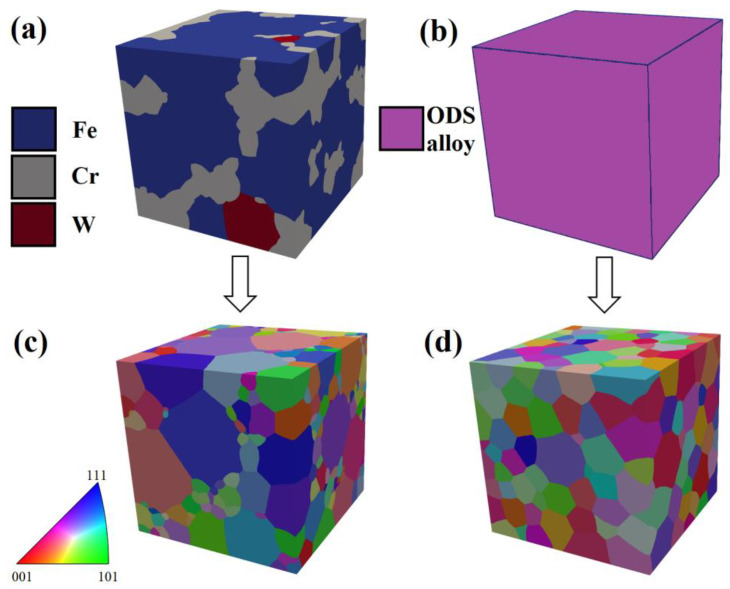
Synthetic microstructures created using WPPM data, visualizing phases detected by XRD after 1 h (**a**) and 64 h (**b**) of MA and domain size distribution after 1 h (**c**) and 64 h (**d**) of MA. The length of the cube edge is 100 nm.

In an effort to visualize the evolution of microstructure in milled powders, a synthetic microstructure was created using domain size statistics obtained by WPPM for 0.5% Zr powder in the DREAM.3D software. According to the XRD analysis, after 1 h of MA, three distinctive phases (Fe, Cr, W) were detected, which later transformed into a single ferritic (bcc) ODS alloy phase after MA (Figure 20a,b). The domain size distribution is visualized in Figure 20c,d. The grains were assumed to be randomly oriented, which is expressed by their random color variation. It can be noticed that much finer domains existed in the gray fields, corresponding to the Cr phase, whereas Fe and W domains (blue and red, respectively) are coarser after 1 h of MA. After 64 h of MA, the visualized microstructure was much more homogenous, consisting of randomly oriented, equiaxed grains with little deviation in size.

**Figure 21:**
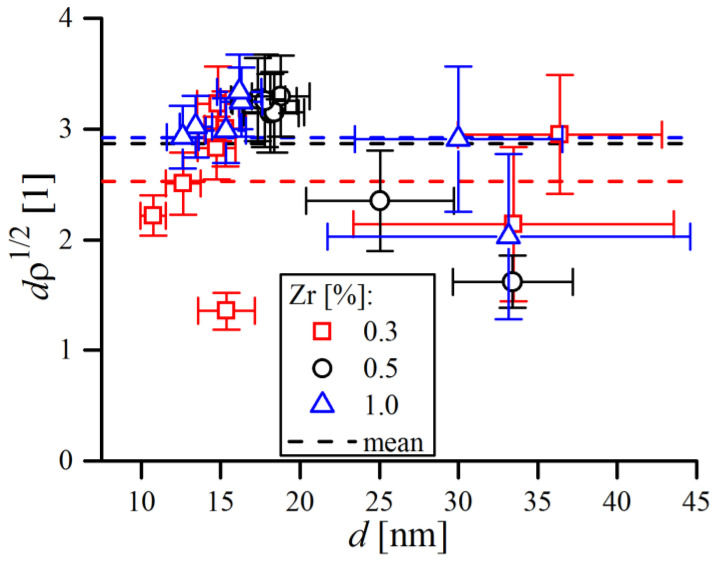
Average number of dislocations per single crystalline domain (ratio between d and $\rho^{-1/2}$) in the function for mean domain diameter. Dashed horizontal lines are average values over entire MA process for a given Zr content.

Severe work hardening of the powder material during grinding was manifested by continuous accumulation of defects in crystals, mainly as dislocations. The evolution of dislocation density (ρ) calculated by WPPM analysis is presented in Figure 18d, which has, in general, very similar trends as the lattice constant. Dislocation density grew during the first 16 h of MA, then the growth ratio slowed dramatically. The final obtained values of ρ are in range of a few times $10^{16}/m^{2}$—4.73(15) $\times 10^{16}/m^{2}$, 3.35(10) $\times 10^{16}/m^{2}$ and 5.38(19) $\times 10^{16}/m^{2}$ for 0.3, 0.5 and 1.0% Zr, respectively, with an effective dislocation cut-off radius $R_{e}$ of ~5 nm. Similar values were reported by other authors, such as Scardi and Leoni [6], Kumar et al. [22] and Rebuffi et al. [24], in the case of FeMo, FeCrAl and Ni ball-milled powders. However, questions may arise about the actual meaning of ρ, as final ρ values were relatively high and seemed overestimated, roughly corresponding to around 2–3 dislocations per single grain (below grain sizes of ~20 nm, sub-grains do not form so crystalline domains can be perceived as grains [46]). The average number of dislocations per grain was calculated as a ratio between the mean grain diameter d and mean dislocation distance $\rho^{-1/2}$—i.e., $d\rho^{1/2}$, and the obtained data points are compiled in Figure 21.

**Figure 22:**
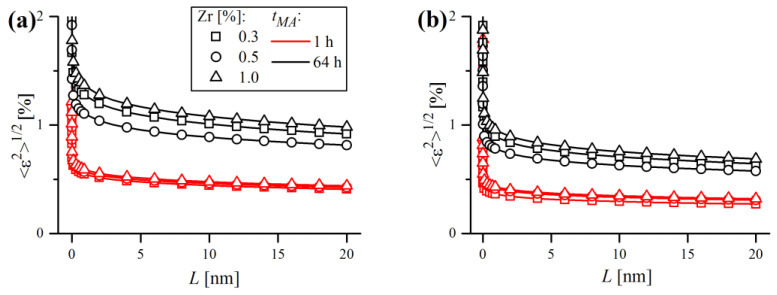
Microstrain plots in function of correlation length for samples after 1 h and 64 h of MA and various Zr content along [h00] (**a**) and [hhh] (**b**) crystallographic directions.

As shown, the average number of dislocations was always above unity, a condition certainly refuted by TEM observations [24] as well as molecular dynamics simulations [47]. Although some dislocation dipoles were found in the TEM observations of ball-milled materials, apparently many crystallites do not contain any dislocations, so the probably strain broadening observed in the XRD line profile has origins that are not solely due to dislocations [48]. In fact, the quantity of dislocations in severely deformed, nanostructured metals is not so high, as dislocations generated during plastic deformation slip across the grains and merge into the grain boundary [24]. Therefore, to avoid reporting ρ values which were clearly overestimated, referring to a more general term of ‘microstrain’ ${〈\varepsilon^{2}〉}^{1/2}$, i.e., root mean square elastic strain, can be more adequate to describe the strain broadening observed in diffraction lines. Microstrain can be simply calculated along any desired crystallographic direction by substitution of elastic strain parameters obtained by WPPM into the equation proposed by Wilkens (Equation (8)):

(8) $${〈\varepsilon_{hkl}^{2}〉}^{1/2}=\left(\rho \bar{C}b^{2}/4\pi \right)f^{*}\left(L/R_{e}\right)$$

where L is the distance between pairs of atoms along the given crystallographic direction (correlation length), $R_{e}$ is the effective dislocation cut-off radius and $f^{*}$ is a monotonically, slowly decreasing logarithmic function, the so-called Wilkens function [49]. Values of ${〈\varepsilon_{hkl}^{2}〉}^{1/2}$ were plotted in Figure 22 for two crystallographic directions, [*h*00] (a) and [hhh] (b), which are the softest and the stiffest crystallographic directions in Fe-bcc, respectively. As expected, microstrain values were higher towards the [h00] direction compared to [*hhh*], thus representing the upper and lower boundaries of the strain broadening.

**Figure 23:**
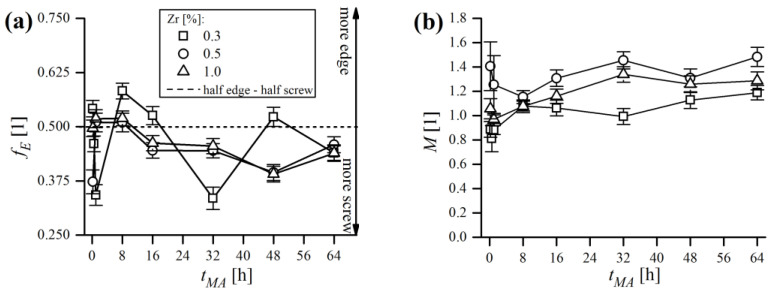
Trends of edge dislocation fraction (**a**) and Wilkens parameter (**b**) during MA of powders.

Furthermore, the dislocation character trend was plotted in the form of the edge dislocation fraction $f_{E}$, which is depicted in Figure 23a. In general, the dislocation structure was in favor of screw dislocations over the whole MA process, as the $f_{E}$ rarely exceeded 0.5, which might be caused by the rotational nature of the ball milling process, promoting the formation of twists in crystals [50]. This is in line with other authors’ conclusions that edge dislocations are unstable in fine Fe domains [24,51].

The inspection of the Wilkens parameter M, was used to determine the correlation of dislocations in the material, i.e., to determine whether the arrangement of dislocations is random or strongly correlated. Its value is also directly correlated with the effective dislocation cut-off radius $R_{e}$, as $M=R_{e}\rho^{1/2}$. It can be determined using XRD LPA, as the peak tail regions decay faster or slower, depending on the weak or strong character of the strain field. When dislocations are randomly distributed, M is large (M≫1). On the contrary, M~1 when dislocations are close to each other, i.e., when the strain field has the short-range character, known as the strong dipole configuration [11]. In the present case (Figure 23b), M values during MA oscillated around unity, finally reaching values in range of ~1.2–1.4 after 64 h of MA, supporting the hypothesis of strong interactions between dislocations, that were observed, for instance, in dislocation cell walls [52]. This indicates that the dislocations were not randomly arranged in crystalline domains, but are rather densely accumulated at grain boundaries. Furthermore, ball milling caused microstructure evolution towards a nanocrystalline state, consisting of fine, narrowly dispersed crystalline domains with strongly correlated extrinsic dislocations forming non-equilibrium grain boundaries.

According to the work hardening theory of cold worked metals, the growth in defects during plastic deformation should be accompanied with corresponding improvements of mechanical properties, e.g., hardness. The Vickers microhardness HV of the powder samples was determined using a digital microhardness tester using two load values: 0.098 N and 0.245 N (10 and 25 g of force, respectively), and 15 s of dwelling time. It was necessary to apply two different load values due to substantial disparities in microhardness between samples tested after short and long MA times. A load of 0.098 N turned out to be too small in the case of harder particles, as the imprints produced were so small that they could not be properly measured. Contrarily, a 0.245 N load was too much for samples after ≤1 h of MA, causing the particles to break apart. As a consequence, powders after short MA times (≤1 h) were tested using a 0.098 N load, and all others used a 0.245 N load. At least 20 indentations were performed on each powder to calculate the mean experimental values of HV.

### 3.6. Microhardness Testing of Mechanically Alloyed Powders

**Figure 24:**
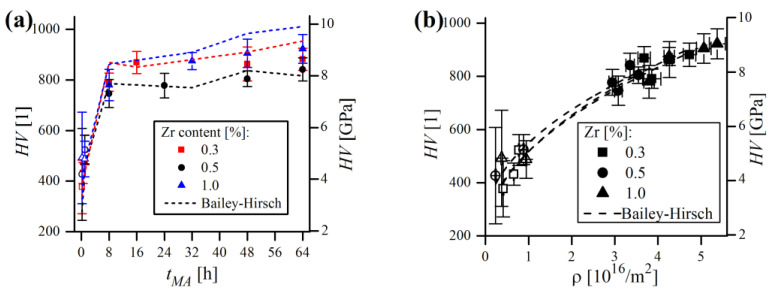
Comparison of experimental (scatter) and model (Equation (11), dashed line) microhardness values of mechanically alloyed powder compositions as a function of MA time (**a**); HV(ρ) relationship (**b**). Hollow points indicate powders tested under 0.098 N load, while filled points indicate a 0.245 N load. Error bars correspond to standard deviation of measurements.

The results of microhardness testing of the mechanically alloyed powders are presented in Figure 24. Powder containing 1% Zr exhibited the highest microhardness after MA (922(56) HV), while 881(44) and 841(46) HV were measured in the case of 0.3% and 0.5% Zr samples, respectively. As seen in Figure 24a, all of them show very similar, exponential trends in HV, with microhardness values raising sharply during the first 8 h of MA, with little increase measured afterwards. At the beginning of MA, the microhardness exhibited the largest variation (expressed in the standard deviation), because the particles were far from being homogenous and were rather complex composites of the starting powders. At this stage, the chemical composition varied significantly from particle to particle and even within the single particle, causing strong dispersion of the obtained HV values. As the process continued, the particles became more homogenous, resulting in lower scattering of measured microhardness.

As the trend of microhardness was, in general, very close to the ρ trend (Figure 24b), and the increase in microhardness itself should result from the accumulation of defects in material, efforts were undertaken to find alleged HV and ρ correlations. The formula relating the material’s strength and dislocation density is basically known as the Bailey–Hirsch relation, originally relating yield strength ($\sigma_{0}$) and ρ [53]. This model assumes that dislocation interaction is the main factor responsible for the hardness increase. However, considering that the hardness of a typical metal is generally 2.5–3 times its yield strength (HV≈2.5 − $3\sigma_{0}$), the following relation, obtained by Yin et al. [54] will be utilized in this work:

(9) $$HV=2.5\sigma_{0}+2.5\alpha Gb\rho^{1/2}$$

where α is a constant (here α = 1) and G is a shear modulus ($G=\left(c_{11}-c_{12}\right)/2$). After grouping the constants by substituting $HV_{0}=2.5\sigma_{0}$ and $k_{HV}=2.5Gb$ one obtains:

(10) $$HV=HV_{0}+k_{HV}\rho^{1/2}$$

Genuinely, the above equation was derived for ball-milled, pure Fe ($HV_{0}=0.363$ GPa, $k_{HV}=60.7\times 10^{-9}\;\mathrm{GPa}\times m)$. In the case of the alloys considered in this work, $HV_{0}$ was estimated as the hardness of pure ferritic Fe, which is ~83.5 HV (0.819 GPa) [55]. The shear modulus was calculated as G = 63 GPa, accordingly to elastic constants reported earlier (Table 1), while b = 0.249 nm, as derived from XRD. After substituting these constants, the HV(ρ) relation can be written as:

(11) $$HV\;\left[\mathrm{GPa}\right]=0.819+39.2\times 10^{-9}\rho^{1/2}\left[\rho \;in\;1/m^{2}\right]$$

or, alternatively, Vickers hardness units can be used, by multiplying the result obtained by a factor of 102 (1 GPa = 102 HV). The first summand in Equation (11) represents the hardness of pure Fe, while the second addend describes dislocation forest hardening. The experimental values of microhardness were compared to those obtained by the model (using ρ values calculated by WPPM) and plotted with relation to MA time in Figure 24a. In any case, HV values obtained from the model mimicked the experimental ones but did not reproduce them clearly. However, modelling the HV(ρ) relationship with Equation (11) (with free $HV_{0}$ and $k_{HV}$ parameters and the ρ exponent constrained to n=1/2) provided a reasonable fit ($R^{2}>0.95$ in all cases), which is presented in Figure 24b. Therefore, the converged $HV_{0}$ and $k_{HV}$ constants derived from trend lines were collated in Table 5.

As shown, $HV_{0}$ values were rather far from the theoretical value of 0.819 GPa, and $k_{HV}$, expected to be around $39.2\times 10^{-9}$ GPa × m tended to be slightly underestimated. The possible reasons for these discrepancies in experimental and modelled HV might be various. First of all, as it was discussed earlier, ρ values obtained from WPPM analysis are probably overestimated. Moreover, the Bailey–Hirsch model might not necessarily describe the macroscopic microhardness evolution in ball-milled materials flawlessly, as it can result not solely from defect accumulation but rather from the combination of collateral effects involved in MA—plausibly related mainly to grain size refinement. As can be noticed in Figure 24a, the theoretical results are understated during early stages of MA (≤1 h) and, in contrast, rather inflated afterwards. Presumably, the major factor contributing to the microhardness of short milled powder is the grain size refinement, which is not accounted for in the Bailey–Hirsch relation, instead of dislocation density, which was low at this stage, causing the theoretical values of HV to be underestimated.

## 4. Conclusions

In this study, the fundamental aspects of mechanical synthesis, mainly in terms of particle size and microstructure evolution, were demonstrated and discussed using the example of three ferritic ODS steel powder compositions. Basic kinematic and energy transfer aspects of the Pulverisette 6 ball mill were characterized by the Magini–Iasonna model. In addition, the quantity of material trapped between the milling balls, ψ ≈ 0.1 mg/mm^2^, was found to be lower than the typical values reported in literature. SEM-EDS and XRD analyses confirmed that around 64 h of MA was required in the presented case to form nanocrystalline and homogenous microstructures.

The microstructural data was extracted by X-ray diffraction LPA, i.e., the WPPM approach. Considering the extensive microstructure analysis with LPA and with the problem of overlapping Fe and Cr reflections, this work presents an important contribution towards this methodology. From the experimental results, the following main conclusions can be drawn: (i) the stored microstrain, expressed in terms of dislocation density, severely increased with milling time and the values tended to saturate in a hyperbolic trend; (ii) crystallite size exhibited progressive decrease to ~15 nm with a little dispersion around the mean diameter and this refinement was especially effective at the initial stages of process; and (iii) the character of dislocation shifted towards the edge dislocation prevalence with milling time.

LPA provided a microstrain value which could be read in terms of strain field from a high density of dislocations (above $10^{16}$ m^−2^) with a short effective cut-off radius ($R_{e}$~5 nm), as an effect of the increased dislocation correlation. Despite the obtained results being within the validity limit of the Krivoglaz–Wilkens theory for the case of strongly interacting dislocations (*M*~1), such a high dislocation density would involve the presence of multiple dislocations in each of the crystalline domains, which is rather unlikely. Hence, the strain broadening observed in the line profiles arose not only from the high strain induced by ball milling, but also from other factors, plausibly concerning the domain boundary aspects. Thus, reporting the microstrain as a function of the correlation length L (root mean square elastic strain) might be more versatile, as it avoids the instability of the Wilkens model, caused by the strong mutual correlation between ρ and $R_{e}$. Moreover, microstrain can be reported along different crystallographic directions, emphasizing the strain field anisotropy of Fe alloys.

## Figures and Tables

**Table 1:** List of essential elastic parameters required to implement strain broadening model.

Element	$a_{0}\;[Å]$	$c_{11}\;[\mathrm{GPa}]$	$c_{12}\;[\mathrm{GPa}]$	$c_{44}\;[\mathrm{GPa}]$	$A_{i}\;$	${\bar{C}}_{h00}^{e}\;$	${\bar{C}}_{h00}^{s}$	$q^{e}$	$q^{s}$
Fe-bcc	2.865	230	134	116	2.41				
Cr	2.884	340	59	99	0.70				
W	3.158	528	193	149	0.89				
Fe-12Cr-2W	2.875	243	117	114	1.82	0.213	0.279	0.712	2.596

**Table 2:** Geometrical dimensions and dependencies of Pulverisette 6 milling equipment used in energy transfer calculations (details in Figure 2 and text).

$r_{d}\;[m]$	$r_{v}\;[m]$	[m]	$r_{b}\;[m]$	$m_{b}\;[g]$	i=ω/Ω
0.061	0.05	0.07	0.005	4.03	−1.82

**Table 3:** Conditions of mechanical alloying applied in this study.

ω [rpm]	Regime	BPR	Milling Media	Atmosphere
300	10 min milling/20 min break	10:1	130 balls; ø10 mm; 4.03 g each	H_2_ (<100 ppb O_2_; <20 ppb H_2_O)

**Table 4:** Overall characteristics of commercial powders used for manufacturing RAF ODS alloys presented in this work. Values in brackets indicate standard deviation of $d_{43}$ measurement.

Element	Purity [%]	Nominal Size	Shape	$d_{43}\;[\mu m]$
Fe	99.9+ *	<10 µm	spherical, rounded	6.9 (1.2)
Cr	99.2 *	<10 µm	spheroidal, plate-like	11.1 (1.3)
W	99.9 *	<44 µm	spherical, multi-faced, rounded	40.7 (0.6)
Zr	98.8 (incl. Hf)	<44 µm	spheroidal, flaky, plate-like	13.2 (0.1)
Y_2_O_3_	99.9	20–40 nm	dendritic agglomerates	N/A

* metals basis.

**Table 5:** Theoretical and recalculated values of $HV_{0}$ and $k_{HV}$ factors from Equation (11). Values in brackets correspond to standard error of fit.

Sample	$HV_{0}\;[\mathrm{GPa}]$	$k_{HV}$ [GPa×m]
Theoretical	0.819	$39.2\times 10^{-9}$
0.3% Zr	1.87(38)	$31.88\left(23\right)\times 10^{-9}$
0.5% Zr	2.69(34)	$28.22\left(22\right)\times 10^{-9}$
1.0% Zr	2.70(48)	$27.08\left(26\right)\times 10^{-9}$

## Data Availability

The data presented in this study are available on request from the corresponding author (K.N.). The data are not publicly available due to privacy.
